# Prognostic signature of esophageal adenocarcinoma based on pyroptosis-related genes

**DOI:** 10.1186/s12920-022-01196-x

**Published:** 2022-03-07

**Authors:** Guo-Sheng Li, Rong-Quan He, Jun Liu, Juan He, Zong-Wang Fu, Lin-Jie Yang, Jie Ma, Li-Hua Yang, Hua-Fu Zhou, Jiang-Hui Zeng, Gang Chen

**Affiliations:** 1 Department of Pathology, The First Affiliated Hospital of Guangxi Medical University, No. 6, Shuangyong Road, Nanning, Guangxi Zhuang Autonomous Region, 530031 China; 2 Department of Medical Oncology, The First Affiliated Hospital of Guangxi Medical University, No. 6, Shuangyong Road, Nanning, Guangxi Zhuang Autonomous Region, 530031 China; 3 Department of Cardiothoracic Surgery, The First Affiliated Hospital of Guangxi Medical University, No. 6, Shuangyong Road, Nanning, Guangxi Zhuang Autonomous Region, 530031 China; 4 Department of Clinical Laboratory, The Third Affiliated Hospital of Guangxi Medical University/Nanning Second People’s Hospital, No. 13 Dancun Road, Nanning, Guangxi Zhuang Autonomous Region, 530031 China

**Keywords:** Adenocarcinoma of esophagus, Pyroptosis, Prognosis, Gene expression

## Abstract

**Background:**

The role of pyroptosis-related genes (PRGs) in esophageal adenocarcinoma (EAC) remains unknown.

**Methods:**

In this study, the first PRGs prognostic signature (PPS) of EAC was constructed based on the results of multivariate stepwise Cox regression analysis. Based on 1,047 samples of EAC and normal esophagus (NE), differentially expressed PRGs were selected for the establishment of the PPS. The discrimination effect of this PPS was detected by receiver operating characteristic curves, and the prognosis value of this PPS was determined through Cox regression analysis and Kaplan-Meier curves. Net benefits of the EAC patients from the nomogram (constructed based on the PPS and some clinical parameters) were assessed via decision curve analysis. The potential molecular mechanism of the PPS in EAC was explored via gene set enrichment analysis. The ability of PPS to distinguish EAC and NE was evaluated based on the results of summary receiver operating characteristic curves.

**Results:**

The significant prognostic value of PPS can be observed at all of the training cohort, test cohort, and validation cohort, such as its independent risk role in the prognosis of the EAC patients (hazard ratio > 0; 95% CI not including 0). The positive net benefits of the nomogram for the EAC patients can be detected via decision curve analysis, and the potential molecular mechanism of the PPS in EAC is likely related to cell pyroptosis. Last, some of the PRGs (particularly *CASP5*) included in this PPS specifically support its feasibility for identifying EAC (area under the curves > 0.7).

**Conclusions:**

The construction of this PPS in EAC enhances the present understanding of the relationship between PRGs and EAC, thus representing a novel approach to the clinical identification and management of EAC based on PRGs.

**Supplementary Information:**

The online version contains supplementary material available at 10.1186/s12920-022-01196-x.

## Background

Esophageal cancer is one of the most common cancers worldwide, ranking 10th in incidence among various other tumors [1]. Globally, the annual estimated number of newly diagnosed cases of and deaths related to esophageal cancer is 604,100 and 544,076, respectively [1]. Clinically, despite the existence of multiple treatment methods, such as surgery, radiotherapy, chemotherapy, and immunotherapy, the 5-year survival probability of esophageal cancer patients is still less than 40% [2, 3], resulting in its ranking sixth among all cancer related-mortality [1]. Esophageal adenocarcinoma (EAC) is one of the two most predominant histopathologies of esophageal cancer, with the other being esophageal squamous cell carcinoma [4]. Specifically, delayed diagnosis is a major factor in the poor prognosis of EAC patients. Unfortunately, most EAC patients are not diagnosed until advanced stages [5], resulting in limited treatment options and lower survival rates. Thus, it is essential to explore novel ideas for the early identification and clinical management of EAC.

The disorder in cell death is one of the essential factors affecting cancer occurrence and progression. Among the numerous different cell death pathways, cell pyroptosis, a pathway initially identified in 2001 by D’Souza et al. [6], is a type of programmed cell death triggered by inflammasomes that, leads to cell swelling, plasma membrane cracking, chromatin fragmentation, and the release of pro-inflammatory substances [7, 8], ultimately, promoting an inflammatory response and activating a strong T cell anti-tumor immune response. Thus, pyroptosis represents one of the new directions of cancer exploration and deserves great potential. Indeed, quite a few pyroptosis-related genes (PRGs) are considered important factors in the proliferation, invasion, and metastasis of several cancers [9]. For instance, in one study, the PRG *NLRP3* was induced by 17β-estradiol, and as a result, it triggered pyroptosis and inhibited the autophagy of hepatocellular carcinoma cells [10]. Also, in another study, the expression of *GSDMC* was found to be promoted by multiple antibiotics such as azithromycin in cancer cells, thereby activating caspase-8 to participate in pyrolysis and leading to the death of breast cancer cells [11]. Another PRG, *GSDME*, is expressed in multiple molecular subtypes of lung cancer, the depletion of which reduces *GSDME*-dependent pyrolysis in non-small-cell lung cancer cells [12]. Therefore, the conclusion can draw that PRGs represent a potential and intriguing application in cancer research. However, little is known about the PRGs in EAC. Thus, this study of EAC in terms of PRGs constitutes a novel perspective, with potential in the clinical identification and management of EAC.

With the progression of biological technologies, such as sequencing technology, the role of prognostic signatures constructed by various genes in tumors has received increasing attention. In this study, based on data from several databases and surveys in the literature, we constructed a PRG prognostic signature (PPS) for EAC. Multiple data sets were used to verify the universality of the signature, and while multi-dimensional analysis was used to evaluate its practicability. In all, the underlying clinical significance of this PPS in EAC is substantially implicated.

## Methods

### Collection of data sets and PRGs

EAC-related data sets were screened and selected from the literature and public databases, including the Gene Expression Omnibus, the Cancer Genome Atlas (TCGA), and the Genotype-Tissue Expression Project (Additional file 1). The search strategy was: “esophag* AND (tumor OR cancer OR carcinoma) AND (mRNA OR gene)”. The inclusion criteria for the data sets consisted of the following: (1) data sets included human esophagus tissues or cells; (2) in each combined data set, for both the EAC group and normal esophagus (NE) group, the number of samples was greater than 3; and (3) mRNA-related data sets (e.g., gene microarrays and RNA-sequencing). Meanwhile, the exclusion criteria consisted of the following: (1) samples derived from non-*homo sapiens*; (2) duplicate or incomplete data; and (3) undefined subtypes of esophageal cancer. In the end, 38 data sets comprised of a total of 1 047 samples (EAC, *n* = 361; NE, *n* = 686) were included in this study (Additional file 2).

For the construction of the PRGs prognostic signature (PPS), the TCGA cohort was randomly divided into a training cohort (comprised of 70% of the samples from the TCGA cohort) and a test cohort (consisting of the other 30%). Samples from the data set GSE19417 (collected from the Gene Expression Omnibus) were used for the external validation cohort. Moreover, the internal validation cohort was composed of the training cohort and the test cohort, as there was not a sufficient number of clinical parameters in the external validation cohort GSE19417.

A total of 33 PRGs were collected from literature retrieval [13]: *AIM2*, *CASP1*, *CASP3*, *CASP4*, *CASP5*, *CASP6*, *CASP8*, *CASP9*, *ELANE*, *GPX4*, *GSDMA*, *GSDMB*, *GSDMC*, *GSDMD*, *GSDME*, *IL18*, *IL1B*, *IL6*, *NLRC4*, *NLRP1*, *NLRP2*, *NLRP3*, *NLRP6*, *NLRP7*, *NOD1*, *NOD2*, *PJVK*, *PLCG1*, *PRKACA*, *PYCARD*, *SCAF11*, *TIRAP*, and *TNF*.

### Data processing

For each data set, the gene expression level was processed with log_2_ (*x* + 1) conversion and normalized with limma package [14] in R (v 4.1.0). The 38 data sets were classified into eight reorganized data sets based on the same platform. For instance, the reorganized data set GPL17692 consisted of GSE74553 and GSE77563 data sets, as these were contained in the same platform: GPL17692 (Additional file 2). The Surrogate Variable Analysis package [15] in R (v 4.1.0) was applied to reduce batch effects between the various datasets, An example of this can be seen in Additional file 3, which shows that: (1) before the batch effects were removed, the samples were clustered according to the dataset rather than the corresponding sample groups (i.e., EAC group, NE group); (2) after the batch effects removed, the samples were no longer distributed according to the data sources, indicating that the main influencing factor in the current sample difference in gene expression levels was the sample group rather than the data source.

### Selection of candidate genes for the PPS

The standardized mean difference (SMD) can eliminate the influence of different measurement units on the results and is especially suitable for the analysis of different numerical data types. In our study, an SMD greater than 0 indicated that a gene had a higher expression level in the EAC group than that in the control group; meanwhile, an SMD less than 0 indicated that the expression level of a gene in the EAC group was lower than that in the control group. An SMD with a 95% confidence interval (CI) not containing 0 or a *p* value less than 0.05 suggested statistical significance. Seventeen of the 33 PRGs were included in both the training cohort and the validation cohort, containing *AIM2*, *CASP1*, *CASP3*, *CASP4*, *CASP5*, *CASP6*, *CASP8*, *CASP9*, *GPX4*, *IL18*, *IL1B*, *IL6*, *PLCG1*, *PRKACA*, *PYCARD*, *TIRAP*, and *TNF*. In terms of the construction of the PPS, among the 17 PRGs, those with SMD values greater than 0 in at least two datasets were identified as upregulated candidate PRG in EAC, while those with SMD values less than 0 in at least two datasets represented downregulated candidate PRGs.

### Construction and validation of the PPS

Based on the candidate PRGs, a multivariate stepwise Cox regression analysis was applied to the training cohort for the construction of the PPS. The risk score for each EAC patient in the training cohort was calculated according to the PPS. The concordance index and area under the curve (AUC) were used to access the probability that the predicted result was consistent with the actual observed result. Both the concordance index and AUC ranged from 0 to 1, the larger these two values, the higher the accuracy of the PPS in predicting the survival probability of the EAC patients. For verification of the PPS, the AUC and calibration curves were also applied to the test cohort and the validation cohort.

### Expression and discrimination effect of PRGs in the PPS

Differential expressions of PRGs in the PPS between the EAC group and the NE group were analyzed in terms of the SMD. Further, the AUC values of summary receiver operating characteristic curves were applied to evaluate the ability of the PRGs from PPS in distinguishing the EAC from the NE group.

### Potential targeted drugs for the PPS

The Connectivity Map (cMap) [16] is a small molecular prediction database. The prediction process is based on differentially expressed genes. The enrichment scores of drug molecules potentially targeting for the PPS can be obtained from cMap; these enrichment scores range from − 1 to 1, with a negative score and a positive score representing that a drug may play an opposite and similar role as the PPS in EAC, respectively. In this study, small molecules with an enrichment score < − 0.8 and *p* value < 0.05 were denoted as potential drugs for the PPS. Through the PubChem database, the three-dimensional structures of potential target drugs for the PPS were obtained.

### Statistical analysis

SMD calculation was performed with meta [17] package in R (v 4.1.0). The relationship between the risk score and clinical parameters—age (in years), gender, tumor stage, node stage, metastasis stage, clinical stage, new event (recurrence or metastasis), Barrett’s esophagus, body mass index—of the EAC patients was determined using a Wilcoxon rank-sum test.

Kaplan–Meier curves and Cox regression analysis were used to explore the performance effect of the PPS on the risk scores of the EAC patients, and these were performed with survival package in R (v 4.1.0). A nomogram was used for calculating the survival probability of the EAC patients. Then, calibration curves were drawn to determine the difference between the predicted and true survival probabilities of the EAC patients. For this nomogram, decision curve analysis was used to determine the net benefits for the EAC patients. In this case, “net benefits” refers to the difference between the benefits and disadvantages of the EAC patients, with larger values indicating more conspicuous benefits the EAC patients obtaining from the nomogram; in other words, the nomogram performed well in predicting survival probabilities in the EAC patients.

Via gene set enrichment analysis (GSEA) conducted in clusterProfiler package in R (v 4.1.0) [18], gene ontology terms (specifically regarding molecular function) and signaling pathways (from both the KEGG [Kyoto Encyclopedia of Genes and Genomes] and Reactome databases) were explored in terms of the potential molecular mechanism of the PPS in EAC. CIBERSORT was applied to analyze the tumor-infiltrating levels of various types of immune cells. Additional file 4 shows the research design of the study.

## Results

### Construction and verification of the PPS

**Fig. 1:**
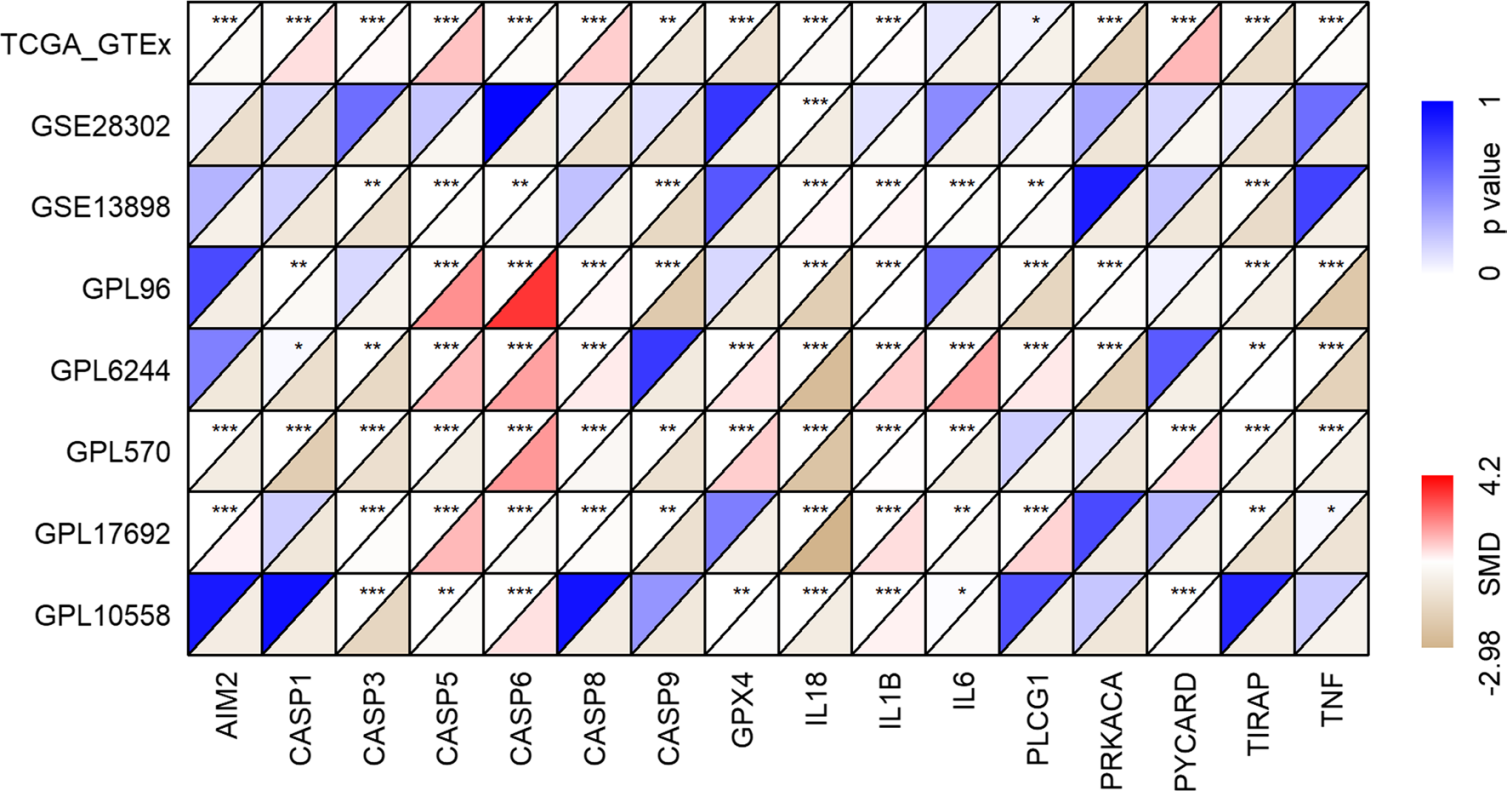
The differerntial expression of 17 pyroptosis-related genes (PRGs) between esophageal adenocarcinoma (EAC) and normal esophagus (NE). SMD, standardized mean difference. *p < 0.05; **p < 0.01; ***p < 0.001

The seventeen PRGs represented in both the training cohort and validation cohort were selected for the construction and verification of the PPS; based on the corresponding SMD results, the 17 PRGs showed either upregulated or downregulated expression in the EAC group in at least two data sets (Fig. 1). The results of Wilcoxon tests also supported the differential expression levels of these 17 PRGs in EAC (Additional files 5, 6). Based on the training cohort, four of the 17 candidate PRGs were applied to construct the PPS based on the results of the multivariate stepwise Cox regression (Table 1). The risk score determined by the PPS for each EAC patient was calculated as 0.3739*CASP5 expression level + 0.9231*CASP8 expression level + 0.3018*IL6 expression level − 0.9970*TIRAP expression level.

**Table 1:** The composition of pyroptosis-related genes (PRGs) prognostic signature

PRGs	Coefficient	Hazard ratio (95% CI)	*p* value
*CASP5*	0.3739	1.4534 (1.0628–1.9875)	0.0192
*CASP8*	0.9231	2.5171 (0.9825–6.4483)	0.0545
*IL6*	0.3018	1.3523 (1.0523–1.7380)	0.0184
*TIRAP*	− 0.9970	0.3690 (0.1800–0.7557)	0.0064

All the values in the table are the results after retaining the four-digit decimal

**Fig. 2:**
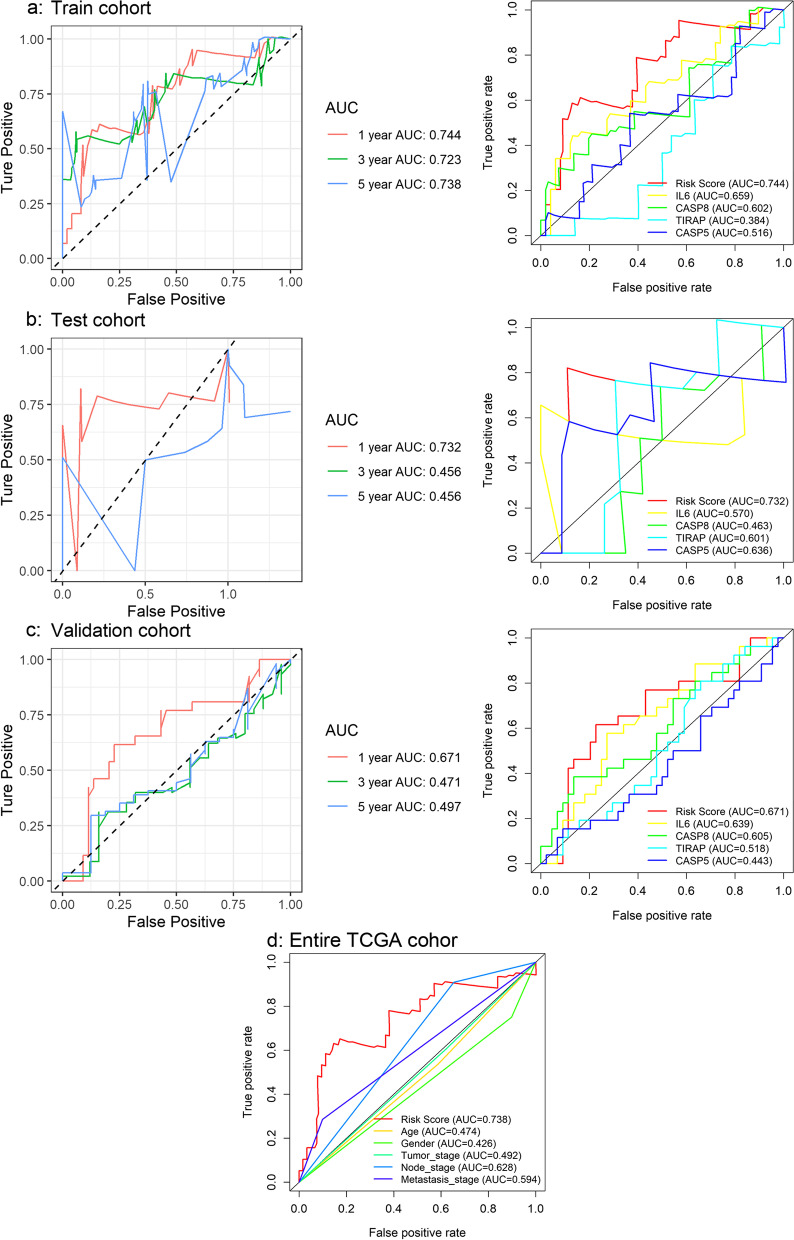
Time-dependent receiver operating characteristic curves based on the PRGs prognostic signature (PPS). In terms of predicting the 1-year survival probability, the risk score of the prognostic signature is more accurate than any single gene in the signature or clinical parameters. **a** The capability of PPS and PRGs in predicting prognosis of EAC patients in the training cohort. **b** The capability of PPS and PRGs in predicting the prognosis of EAC patients in the validation cohort. **c** The capability of PPS and PRGs in predicting the prognosis of EAC patients in the test cohort. **d** The capability of PPS and clinical parameters in predicting the prognosis of the EAC patients in the entire TCGA cohort

The predicted survival probability of the EAC patients determined by the PPS was close to the actual survival rate, as the concordance index in the training cohort was 0.682 (data not shown). Such a conclusion can also be drawn based on the AUC value (> 0.74) of predicted 1-year survival probability based on the training cohort; and further, the AUC values of both the test cohort and validation cohort were also > 0.67, although the risk score showed a poorer effect in predicting the 3- and 5-year survival probability for the EAC patients (Fig. 2a–c). Moreover, the risk score of the prognostic signature was more accurate than any PRGs (Fig. 2a–c) in the PPS and any clinical parameters alone (Fig. 2d) in predicting the 1-year survival probability. Thus, the ability of PPS to predict the prognosis of the EAC patients can be confirmed.

### Relationship between risk score and clinical parameters

**Fig. 3:**
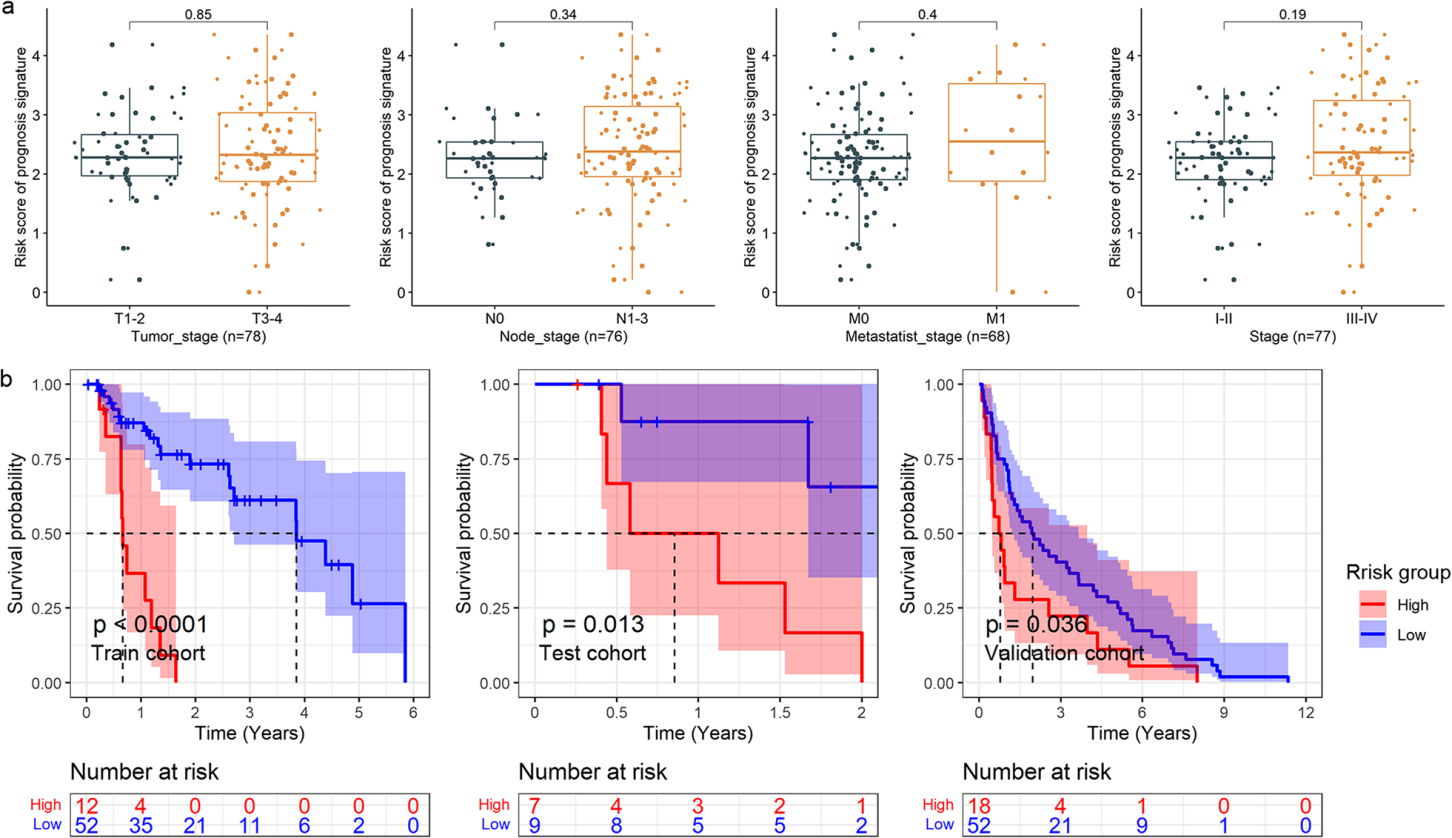
Box plots and Kaplan–Meier curves. **a** The relationship between the prognostic signature and clinical parameters of EAC patients. The *p* value at the top for each panel is based on the Wilcoxon test. **b** Kaplan–Meier curves of high- and low-risk groups

No clinical parameters (e.g., age, gender, tumor stage, node stage, metastasis stage, clinical stage, new event, Barrett’s esophagus, body mass index) of the EAC patients were found to be related to their respective risk score. However, an associating trend between a high risk score of the PPS with higher tumor stage, node stage, metastasis stage, and clinical stage can be observed (Fig. 3a).

### Prognostic significance of the PPS

**Fig. 4.**
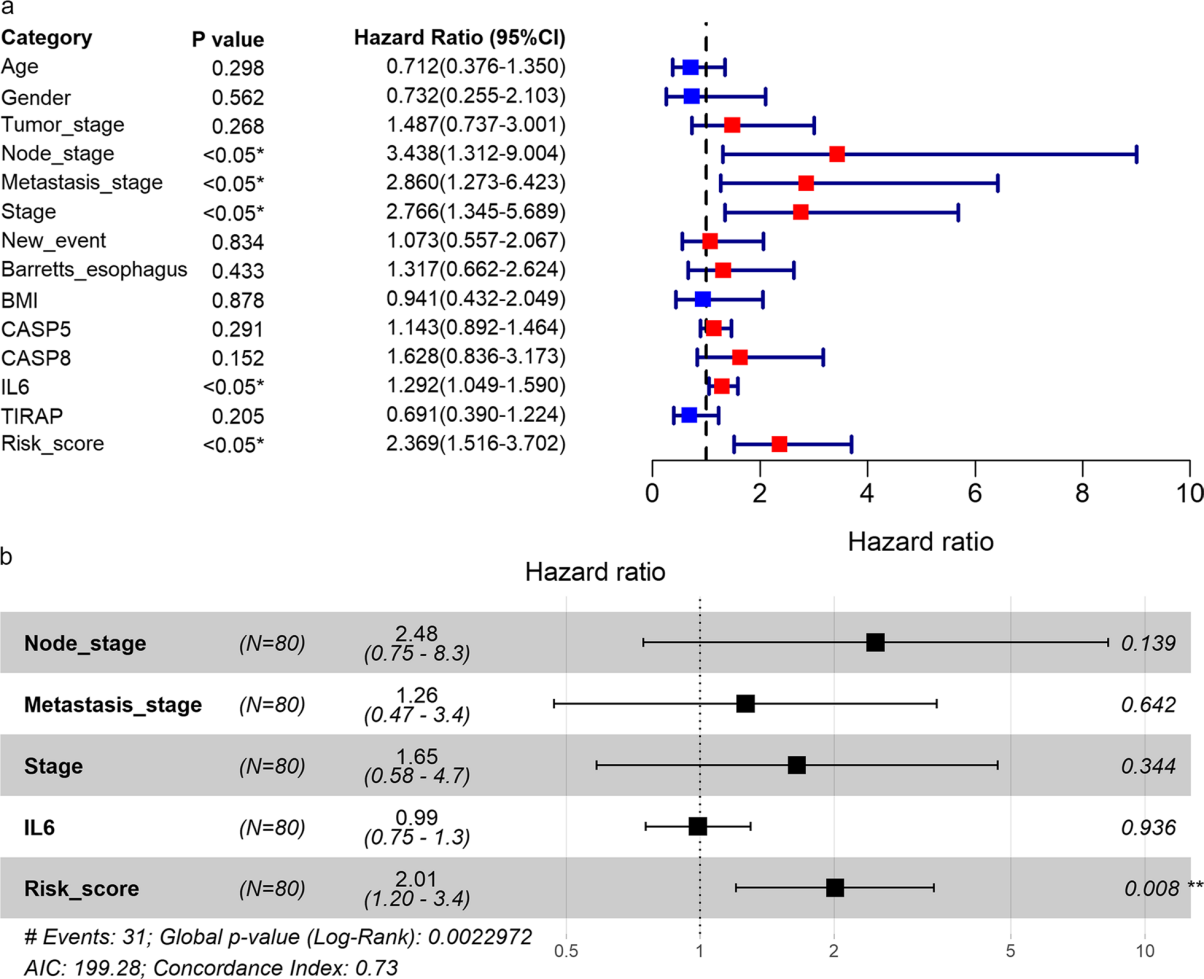
Identification of prognostic factors. Forest plots for univariate (**a**) and multivariate (**b**) Cox regression analyses of high- and low-risk groups in terms of the detection by the PPS and clinical parameters of EAC patients

In the group of patients with higher risk scores, a higher number of deaths can be observed than the group with lower risk scores (Additional file 7). For the training cohort, test cohort, and validation cohort, the risk score of the PPS was related to a shorter survival time of the EAC patients (*p* < 0.05, Fig. 3b). Furthermore, based on the internal validation cohort (consisting of the training cohort and test cohort), univariate (Fig. 4a) and multivariate (Fig. 4b) Cox regression analyses suggested that the risk score, rather than the tumor stage, node stage, metastasis stage, or *IL6* expression, was an independent risk factor for poor prognosis in the EAC patients (hazard ratio > 1; 95% CI not including 1).

### Construction and verification of nomogram

**Fig. 5:**
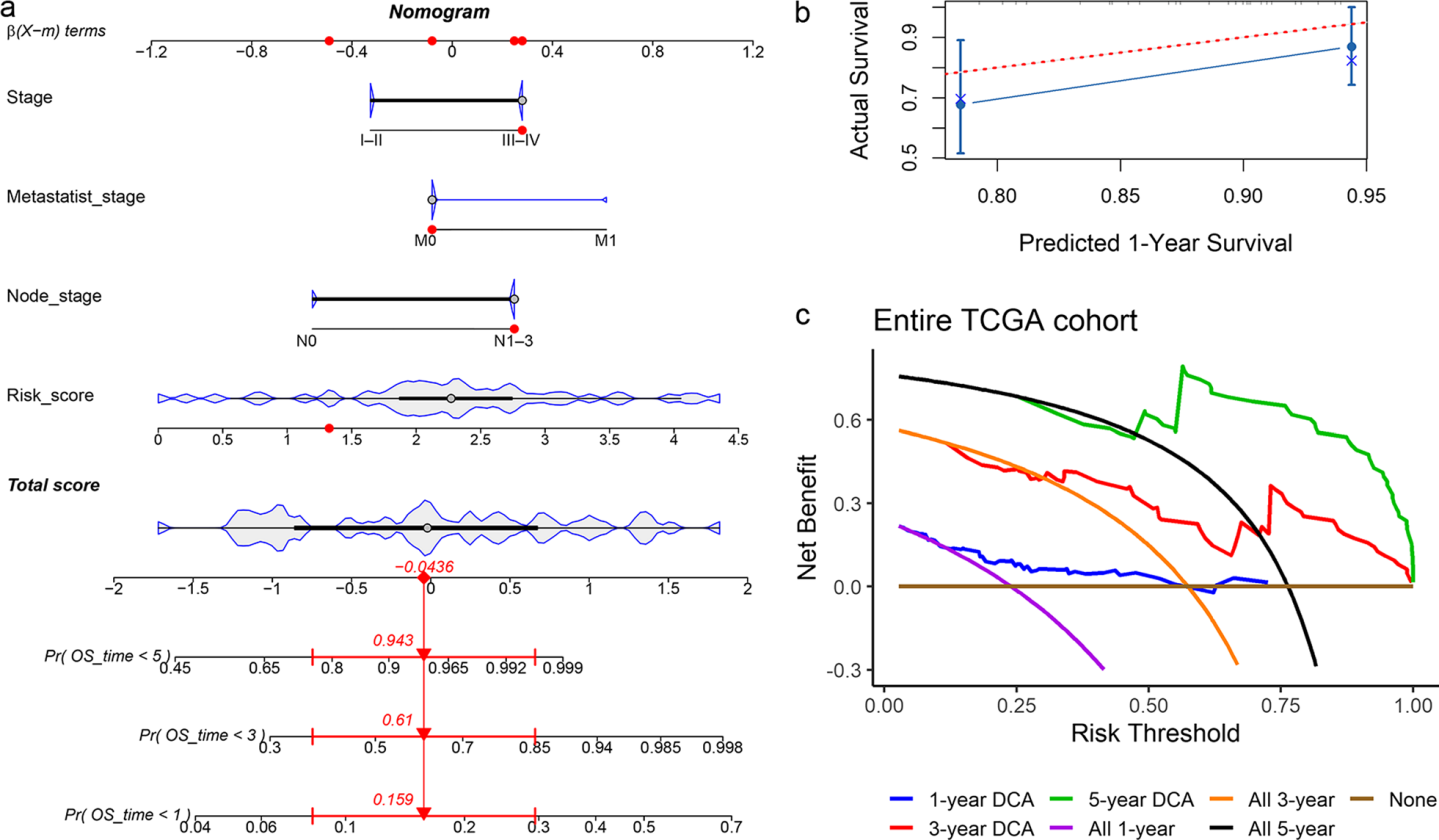
Nomogram and its verifications. **a** Nomogram for predicting prognosis of EAC patients; the red dots represent the clinical features of one EAC patient, and, as predicted, his chances of surviving fewer than 5 years, 3 years, and 1 year were 0.943, 0.61, and 0.159, respectively. **b** Calibration curve of the nomogram. **c** Decision curve analysis (DCA) of the nomogram

Considering the prognostic value of the PPS in the EAC patients, we established a nomogram based on the risk score and the clinical parameters with *p* < 0.05 in univariate Cox regression analysis (Fig. 4a), as shown in Fig. 5a. The 1-year survival rate predicted by the nomogram fluctuated slightly varied the patient’s actual 1-year survival probability (Fig. 5b), while lesser prediction effects of the PPS were observed for the EAC patients’ 3-year and 5-year survival rates (data now shown). For the nomogram, positive net benefits for the EAC patients were detected via decision curve analysis (Fig. 5c).

### Underlying molecular mechanism of the PPS in EAC

**Fig. 6:**
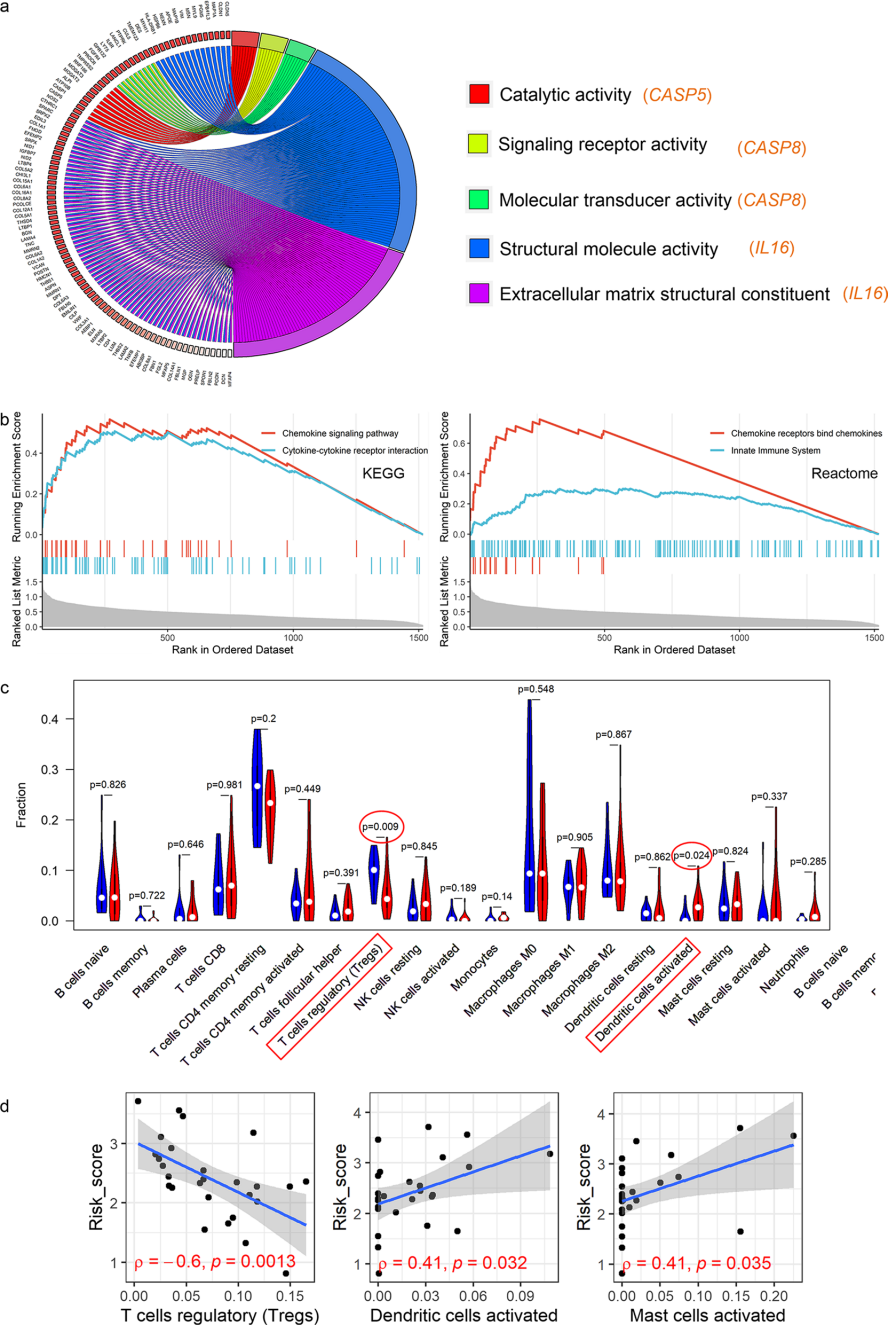
GSEA and immune correlation analyses. **a** The molecular functions where three PRGs may participate. **b** The high-risk group is enriched in the gene set related to immune response. **c** The rate of immune cell infiltration between the high-risk group and low-risk group based on the prognostic signature. **d** The correlation of the risk score with three immune cells; *ρ*, Spearman’s coefficient

Some molecular functions of the PRGs of the PPS were investigated in EAC based on GSEA. As a result, there was at least one molecular function term reaching statistical significance for *CASP5*, *CAPS8*, and *IL6*, but this was not true for *TIRAP* (Additional file 8). Specifically, the GSEA indicated the following: *CASP5* may participate in catalytic activity (broad enzyme activity); *CAPS8* may be related to signaling receptor activity and molecular transducer activity; *IL6* may play roles in EAC by effecting structural molecule activity and extracellular matrix structural constituents (Fig. 6a).

Based on the GSEA, genes in the high-risk group tended to cluster in some KEGG and Reactome signaling pathways related to immune response, such as innate immunity pathways (Fig. 6b). Thus, we further explored the relationship between the risk score and immune cell infiltration levels for each EAC sample (Additional file 9). EAC patients with elevated risk scores were observed with lower infiltration levels of regulatory T cells and higher infiltration levels of activated dendritic cells (Fig. 6c). Consistent with this, correlation analyses also demonstrated a negative association between the risk score and regulatory T cells as well as a positive relevance of the risk score with activated dendritic cells (Fig. 6d). Furthermore, EAC patients with increased resting mast cells as well as CD8^+^ T cell infiltration levels tended to have a longer survival time (*p* value < 0.05) (Additional File 10). The risk score was also positively correlated with activated mast cells (Fig. 6d), while no statistically significant difference in the infiltration levels of the activated mast cells between the high-risk group and low-risk group was found (Fig. 6c).

### Expression and discrimination effect of PRGs in PPS

**Fig. 7:**
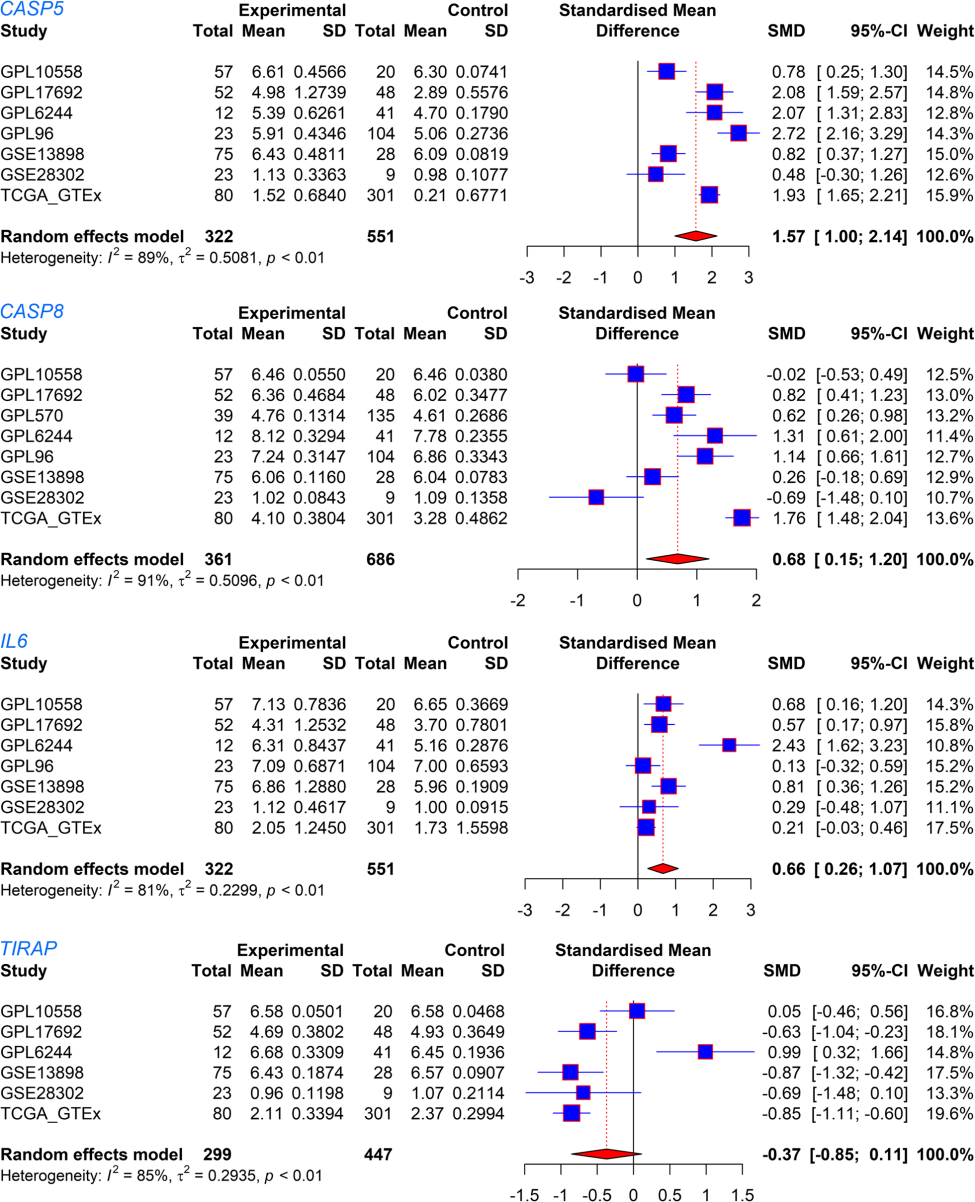
Forest plots of the expressions of the PRGs. Expression differences in *CASP5*, *CASP8*, *IL6*, and *TIRAP* between the EAC group and NE group can be observed

**Fig. 8:**
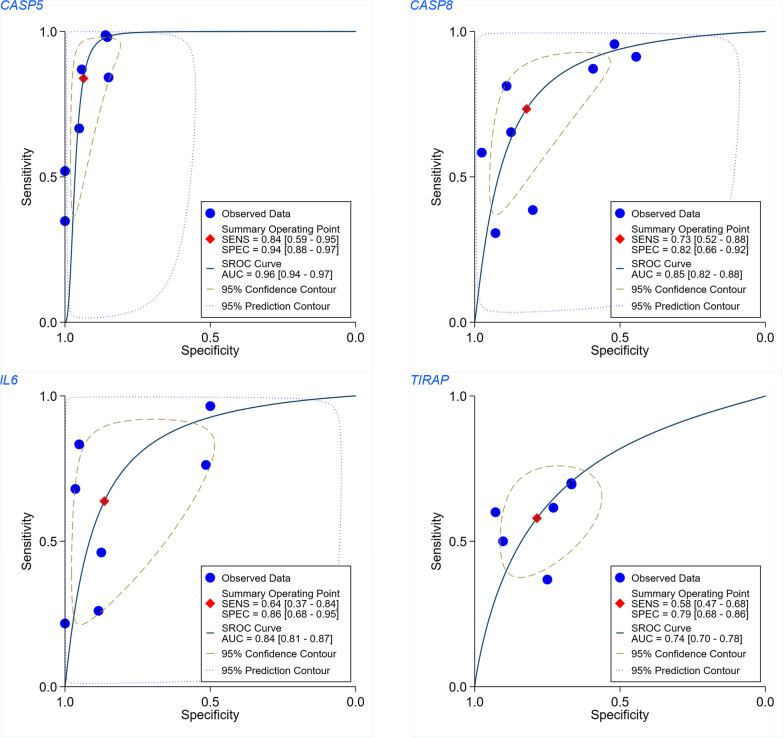
Summary receiver operating characteristic curves evaluating whether the four PRGs can identify esophageal adenocarcinoma

The differential expression of the four PRGs—*CASP5*, *CASP8*, *IL6*, and *TIRAP*—between the EAC and NE groups can be observed in Fig. 7. Further, the AUC values for these four PRGs in terms of distinguishing EAC from NE were all > 0.74; for *CASP5*, the AUC value reached 0.96, indicating this gene’s particular ability in distinguishing EAC from NE (Fig. 8).

### Potential target drugs for PPS

Based on the analysis in the cMap database, altizide and harpagoside were the two small molecules identified as potential target drugs for the PPS with enrichment scores < − 0.8 (*p* < 0.05). The two-dimensional and three-dimensional structures of altizide and harpagoside can be seen in Additional file 11.

## Discussion

The reduction of cancer cell death is one of the important mechanisms leading to cancer deterioration. Cancer cell death includes apoptosis, autophagy, and pyroptosis [9], all of which represent trendy directions for exploring the mechanism of tumor suppression. Although apoptosis and other cell death methods have been extensively studied, little is known about the relationship between pyroptosis and cancers including EAC.

In this study, using multiple statistical methods, we constructed a reliable signature for EAC based on specific PRGs. Overall, not only were the differentially expressed PRGs selected based on quite a few samples, but the universality of the PPS was supported in the training cohort, test cohort, and validation cohort. As far as we know, this PPS is the first EAC-related prognostic signature based on PRGs. Ultimately, the significant prognostic value of this PPS can be observed in the present study, including its independent risk-prediction role in the prognosis of EAC patients. Further, we investigated the potential molecular mechanism of the PPS in EAC by exploring the underlying reasons for its good prognostic performance in identifying EAC. Specifically, the PPS’s clinical significance terms of prediction performance and outstanding EAC discrimination ability of its PRGs. In this study, the potential target drugs for the PPS were also predicted. In sum, the construction of this PPS in EAC has helped further the understanding of the relationship between PRGs and EAC, representing a novel attempt at the clinical identification and management of EAC in the aspect of PRGs.

The prognostic signature—PPS—constructed by us has noticeable potential clinical value. Prognostic signatures have been extensively studied in various cancers. For example, Wei et al. [19] constructed a bladder cancer-related prognostic signature based on immune-related genes that exhibited a remarkable ability to predict patients’ prognosis. Also, the glycolysis-related signature established by Dai et al. [20] has been well applied in predicting breast cancer survival. In the present study, we constructed a novel PPS, that outperformed each PRG and several clinical parameters in terms of prognostic prediction ability. Moreover, as an independent prognostic risk factor, the PPS-based risk score can be used to preliminarily classify EAC patients and has application potential in clinical practice. For instance, the timely identification of high-risk patients by the PPS could lead to earlier treatment measures for affected patients and ultimately improve the survival rate of EAC patients. For patients at low risk, the temporary deferral of treatment may be considered to avoid unnecessary treatment and reduce treatment costs.

The four PRGs—*CASP5*, *CASP8*, *IL6*, and *TIRAP*—in this PPS were found to be closely associated with the development of cancers. Specifically, *CASP5* may play different roles in various cancers. As one of the main mediators of apoptosis, *CASP5* was identified as a potential marker for the treatment of cancer. For instance, *CASP5* expression was related to favorable overall survival in gastric cancer [21]. However, not such a protective role but instead a risk effect has been observed in clear cell renal cell cancer [22]. While as far as we know, this is the first study to find upregulated *CASP5* expression as well as its excellent discrimination effect in EAC, the protective role or risk effect of *CASP5* expression in EAC patients remains unclear and requires further research with multiple samples. Additionally, the elevated expression of *CASP8* and its identifying effect in EAC were detected in our study. Wu et al. reported that rs1035142 (a polymorphism of *CASP8*) was relevant in the early occurrence of EAC [23], and its association with poor prognosis in gastric cancer patients was identified by Wang et al. [21]. High- *IL6* mRNA expression has also been seen in EAC, and unlike for *CASP5* and *CASP8*, there are quite a few studies on this gene in EAC. For instance, *IL6* was found to promote the growth of EAC cells [24]; additionally, it was observed to lead to the enhancement of the therapeutic resistance, migration ability, and proliferation ability of EAC cells, which may be related to the process of activated epithelial-mesenchymal transition [25]. Considering the ability of *IL6* to identify EAC based on AUC and as well as the fact that it has been detected in blood [24, 25], *IL6* expression represents promising screening potential in EAC patients in the future. In terms of *TIRAP*, another PRG in the PPS, it was associated with the proliferation of non-small-cell lung cancer cells, while the knockdown of its expression was found to inhibit cell proliferation [26]. Moreover, *TIRAP* p. R81C was found to promot the proliferation of lymphoma cells, which may be caused by NF-κB-mediated signal transduction in B cells [27]. In summary, the four PRGs of the PPS have clinical significance in a variety of tumors, but their clinical role in EAC and the mechanisms of this disease require further research.

PPS may play its role in EAC by taking part in the immune response. In this study, via GSEA, the potential molecular functions of three PRGs—*CASP5*, *CAPS8*, and *IL6*—in EAC were investigated (no statistical significance was found for *TIRAP*). Overall, *CASP5* may contribute to catalytic activity; *CAPS8* was involved in signaling receptor activity and molecular transducer activity; *IL6* may take part in the structural molecule activity and extracellular matrix structural constituents. Furthermore, immune-related signaling pathways were observed in high-risk groups. Also, it can be detected an association of elevated infiltration levels of activated dendritic cells were with the high-risk group based on the risk score determined by PPS. Dendritic cells can secrete a variety of chemokines, which can be recognized by CD8^+^ T cells, resulting in CD8^+^ T cell infiltration [28]. Moreover, CD8^+^ T cells are thought to inhibit tumor progression by inducing pyroptosis [29]. Interestingly, in our study, EAC patients with high CD8^+^ T cell infiltration levels exhibited a more favorable prognosis than those with low CD8^+^ T cell infiltration levels. Therefore, according to the present study’s results, we speculate that the potential molecular mechanism of PPS in the prognosis of EAC may be due to its classical function—cell pyroptosis; however, this requires experimental verification.

Last, although this PPS shows potential clinical value in EAC, some limitations of the research can also be observed. First, the clinical parameters included in this study were relatively limited. For example, resulting from a lack of data, we failed to determine the relationship between environmental factors (e.g., smoking) of EAC patients and the PPS. Second, the PPS was constructed only from PRGs collected from the literature survey. Third, prospective data are needed to verify: (1) the correlation of the PRGs with the lower infiltration levels of the regulatory T cells and higher infiltration levels of the activated dendritic cells, and, (2) the clinical significance of the PPS and its nomogram.

## Conclusions

In summary, this study constructed a PPS with significant prognosis and differential clinical value, thus indicating its potential in the identification and personalized management of EAC patients.

## Supplementary Information

**Additional file 1**. Collection of data sets for the study.

**Additional file 2**. The data sets included in this study.

**Additional file 3**. The distribution of the reorganized data set GPL17692 before reducing the batch effects is scattered; while after the batch effects are removed, the distribution is well clustered.

**Additional file 4**. The research design of the study. PRGs, pyrolysis-related genes; PPS, pyrolysis-related prognostic signature; EAC, esophageal adenocarcinoma.

**Additional file 5**. Violin and box plots for comparing the differential expression levels of PRGs between the EAC groups and the NE groups. NS,* p* > 0.05; *p < 0.05; **p < 0.01; ***p < 0.001.* p* values are based on Wilcoxon tests.

**Additional file 6**. Violin and box plots for comparing the differential expression levels of PRGs between the EAC groups and the NE groups. NS,* p* > 0.05; *p < 0.05; **p < 0.01; ***p < 0.001.* p* values are based on Wilcoxon tests.

**Additional file 7**. Risk plots and heatmaps. Risk plots for high- and low-risk groups. Each blue dot represents a living patient, while each red dot represents a dead patient. Heatmap of expression levels of the four pyrolysis-related genes between high- and low-risk groups.

**Additional file 8**. GSEA results for* CASP5*,* CAPS8*, and* IL6* in this study.

**Additional file 9**. Immune cell infiltration in high-risk and low-risk groups for each sample.

**Additional file 10**. Esophageal adenocarcinoma patients with mast cells resting or CD8+ T cells infiltration level tend to have longer survival time.

**Additional file 11**. The two-dimensional and three-dimensional structures of potential drugs for pyrolysis-related genes prognostic signature – altizide and harpagoside.

## Data Availability

The datasets supporting the conclusions of this article are available in the Gene Expression Omnibus [GSE28302, https://www.ncbi.nlm.nih.gov/geo/query/acc.cgi?acc=GSE28302; GSE72873, https://www.ncbi.nlm.nih.gov/geo/query/acc.cgi?acc=GSE72873; GSE93352, https://www.ncbi.nlm.nih.gov/geo/query/acc.cgi?acc=GSE93352; GSE47763, https://www.ncbi.nlm.nih.gov/geo/query/acc.cgi?acc=GSE47763; GSE57130, https://www.ncbi.nlm.nih.gov/geo/query/acc.cgi?acc=GSE57130; GSE74553, https://www.ncbi.nlm.nih.gov/geo/query/acc.cgi?acc=GSE74553; GSE77563, https://www.ncbi.nlm.nih.gov/geo/query/acc.cgi?acc=GSE77563; GSE92396, https://www.ncbi.nlm.nih.gov/geo/query/acc.cgi?acc=GSE92396; GSE100843, https://www.ncbi.nlm.nih.gov/geo/query/acc.cgi?acc=GSE100843; GSE34619, https://www.ncbi.nlm.nih.gov/geo/query/acc.cgi?acc=GSE34619; GSE36725, https://www.ncbi.nlm.nih.gov/geo/query/acc.cgi?acc=GSE36725; GSE37200, https://www.ncbi.nlm.nih.gov/geo/query/acc.cgi?acc=GSE37200; GSE1420, https://www.ncbi.nlm.nih.gov/geo/query/acc.cgi?acc=GSE1420; GSE13083, https://www.ncbi.nlm.nih.gov/geo/query/acc.cgi?acc=GSE13083; GSE52138, https://www.ncbi.nlm.nih.gov/geo/query/acc.cgi?acc=GSE52138; GSE23400, https://www.ncbi.nlm.nih.gov/geo/query/acc.cgi?acc=GSE23400; GSE44021, https://www.ncbi.nlm.nih.gov/geo/query/acc.cgi?acc=GSE44021; GSE13898, https://www.ncbi.nlm.nih.gov/geo/query/acc.cgi?acc=GSE13898; GSE26886, https://www.ncbi.nlm.nih.gov/geo/query/acc.cgi?acc=GSE26886; GSE64894, https://www.ncbi.nlm.nih.gov/geo/query/acc.cgi?acc=GSE64894; GSE42363, https://www.ncbi.nlm.nih.gov/geo/query/acc.cgi?acc=GSE42363; GSE9974, https://www.ncbi.nlm.nih.gov/geo/query/acc.cgi?acc=GSE9974; GSE7964, https://www.ncbi.nlm.nih.gov/geo/query/acc.cgi?acc=GSE7964; GSE7307, https://www.ncbi.nlm.nih.gov/geo/query/acc.cgi?acc=GSE7307; GSE148247, https://www.ncbi.nlm.nih.gov/geo/query/acc.cgi?acc=GSE148247; GSE45670, https://www.ncbi.nlm.nih.gov/geo/query/acc.cgi?acc=GSE45670; GSE27424, https://www.ncbi.nlm.nih.gov/geo/query/acc.cgi?acc=GSE27424; GSE17353, https://www.ncbi.nlm.nih.gov/geo/query/acc.cgi?acc=GSE17353; GSE3526, https://www.ncbi.nlm.nih.gov/geo/query/acc.cgi?acc=GSE3526; GSE19472, https://www.ncbi.nlm.nih.gov/geo/query/acc.cgi?acc=GSE19472; GSE33810, https://www.ncbi.nlm.nih.gov/geo/query/acc.cgi?acc=GSE33810; GSE100942, https://www.ncbi.nlm.nih.gov/geo/query/acc.cgi?acc=GSE100942; GSE17351, https://www.ncbi.nlm.nih.gov/geo/query/acc.cgi?acc=GSE17351; GSE77861, https://www.ncbi.nlm.nih.gov/geo/query/acc.cgi?acc=GSE77861; GSE161533, https://www.ncbi.nlm.nih.gov/geo/query/acc.cgi?acc=GSE161533], the ArrayExpress [https://www.ebi.ac.uk/arrayexpress/], TCGA Research Network [www.cancer.gov/tcga], and Genotype Tissue Expression [https://commonfund.nih.gov/GTEx].

## Abbreviations

EAC: Esophageal adenocarcinoma
PRG: Pyroptosis-related gene
PPS: Pyroptosis-related genes prognostic signature
TCGA: The Cancer Genome Atlas
NE: Normal esophagus
SMD: Standardized mean difference
AUC: Area under the curve
cMap: Connectivity Map
GSEA: Gene set enrichment analysis
