# Supplementary figures and images for: Prognostic signature of esophageal adenocarcinoma based on pyroptosis-related genes
Source: BMC Med Genomics. 2022 Mar 7;15:50. doi: 10.1186/s12920-022-01196-x (PMC8900411; doi:10.1186/s12920-022-01196-x)

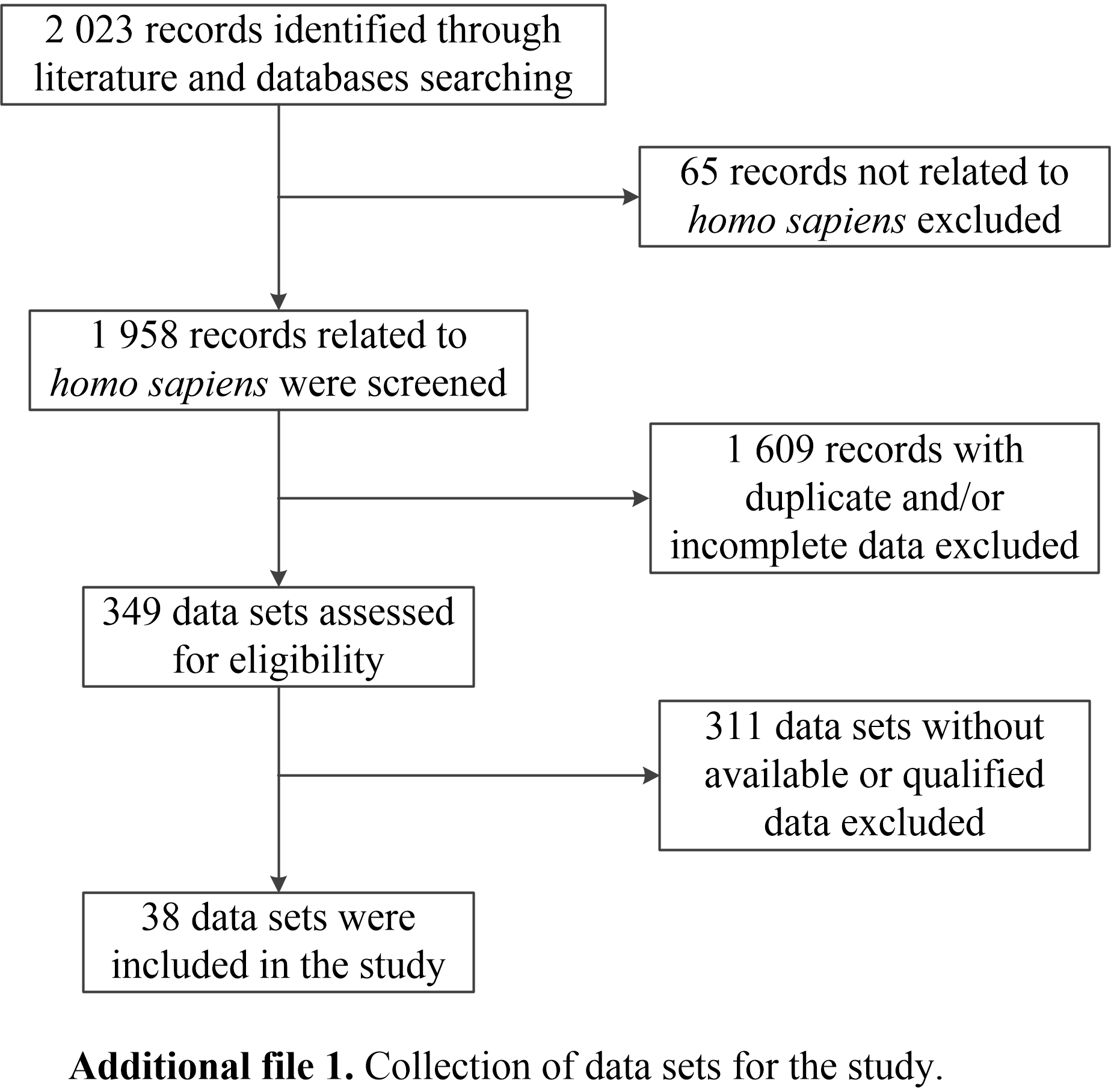

Supplement: Supplementary file 1 — Additional file 1. Collection of data sets for the study. [file 12920_2022_1196_MOESM1_ESM.tif]

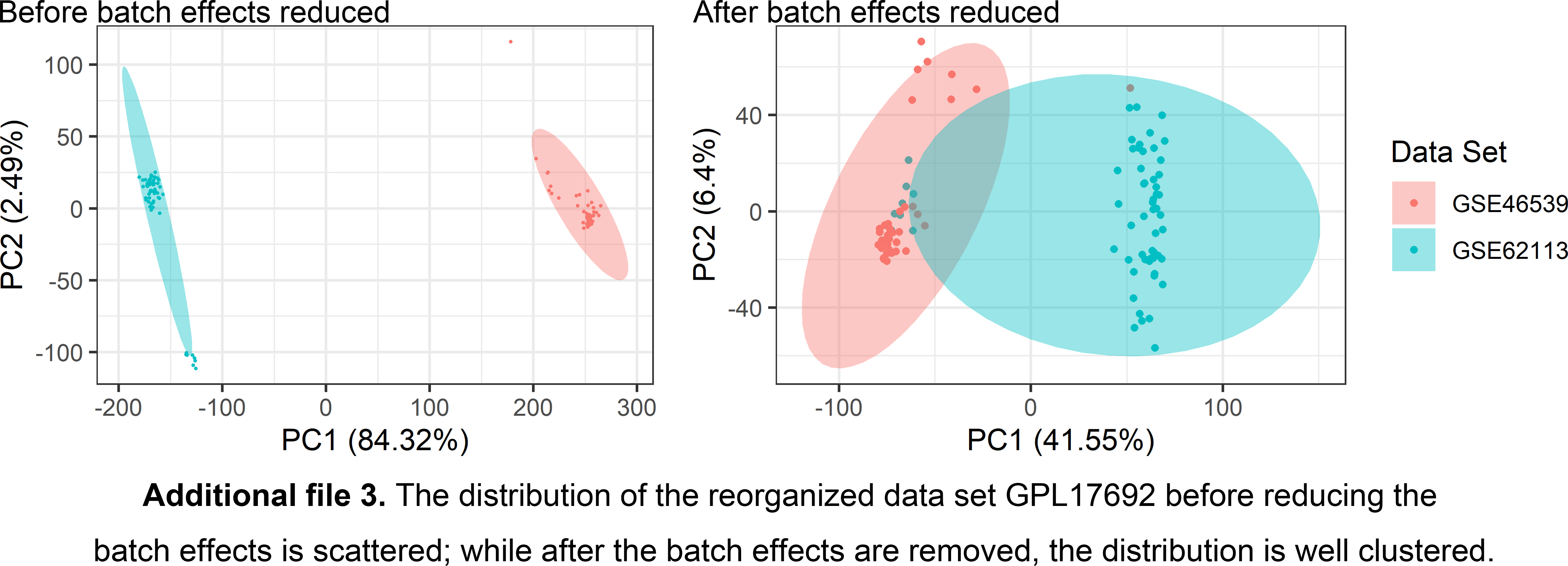

Supplement: Supplementary file 3 — Additional file 3. The distribution of the reorganized data set GPL17692 before reducing the batch effects is scattered; while after the batch effects are removed, the distribution is well clustered. [file 12920_2022_1196_MOESM3_ESM.tiff]

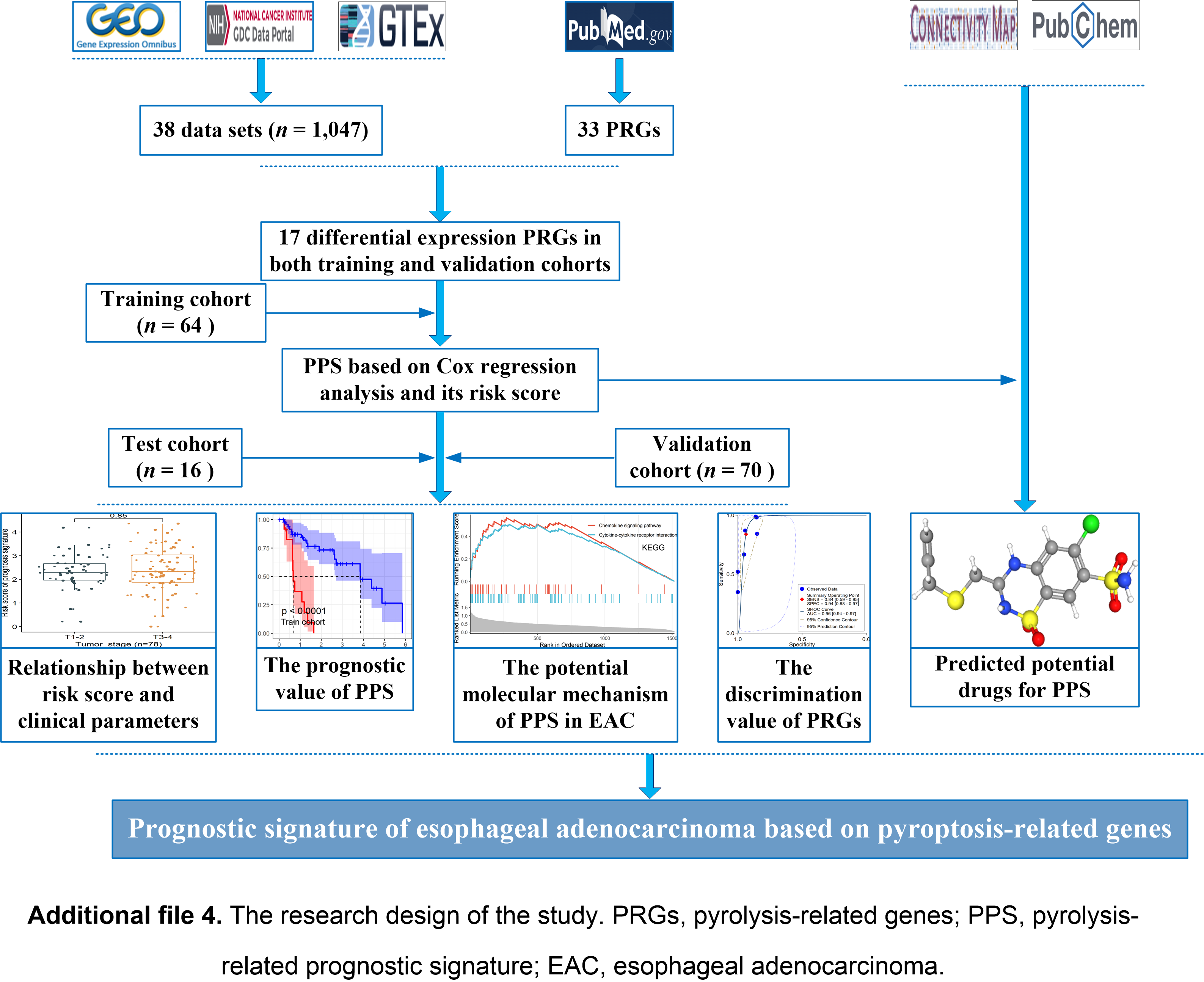

Supplement: Supplementary file 4 — Additional file 4. The research design of the study. PRGs, pyrolysis-related genes; PPS, pyrolysis-related prognostic signature; EAC, esophageal adenocarcinoma. [file 12920_2022_1196_MOESM4_ESM.tif]

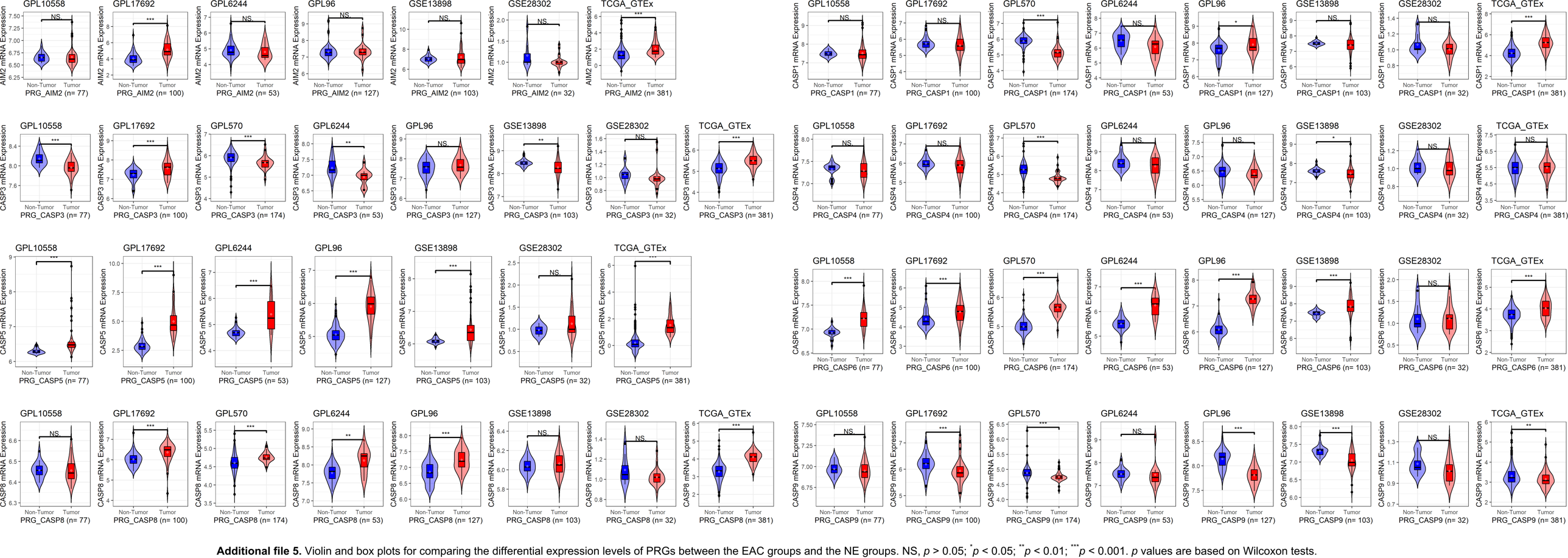

Supplement: Supplementary file 5 — Additional file 5. Violin and box plots for comparing the differential expression levels of PRGs between the EAC groups and the NE groups. NS, p > 0.05; *p < 0.05; **p < 0.01; ***p < 0.001. p values are based on Wilcoxon tests. [file 12920_2022_1196_MOESM5_ESM.tif]

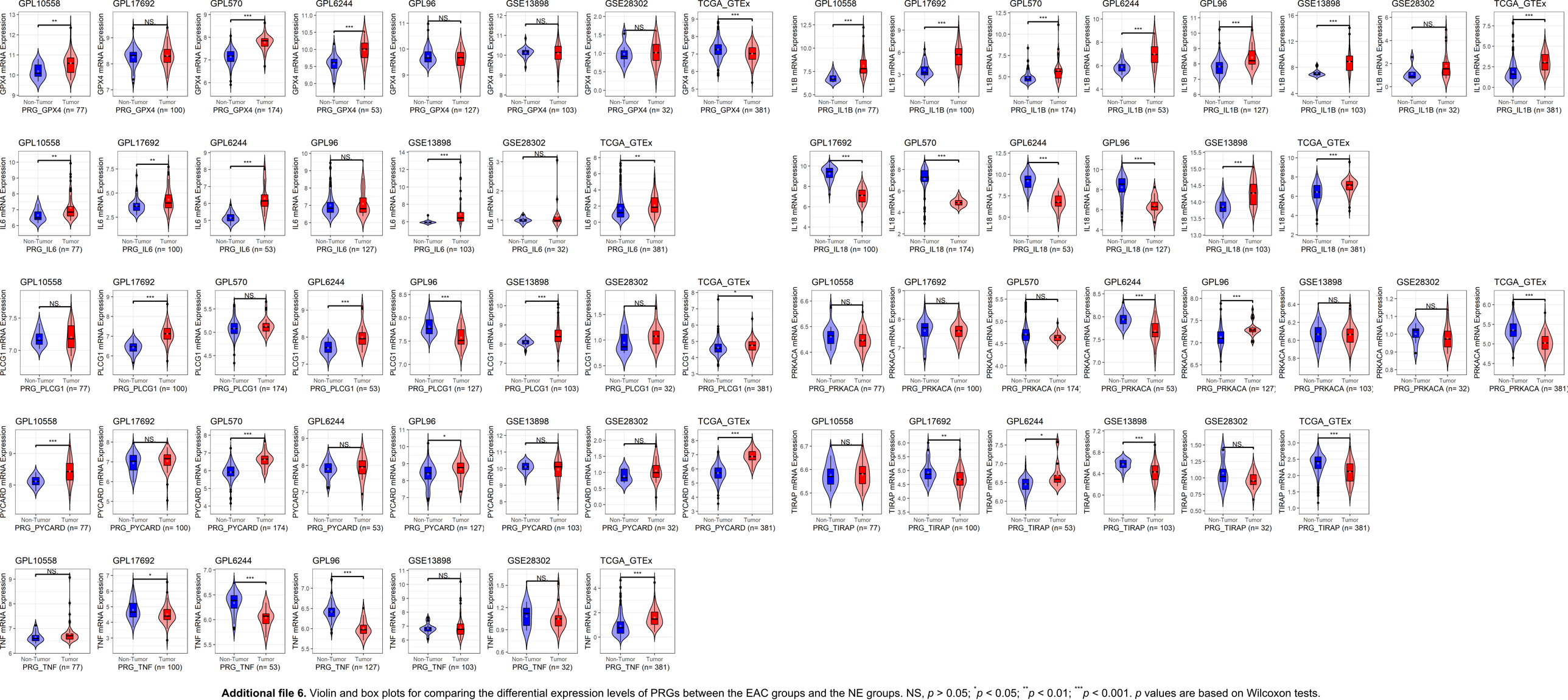

Supplement: Supplementary file 6 — Additional file 6. Violin and box plots for comparing the differential expression levels of PRGs between the EAC groups and the NE groups. NS, p > 0.05; *p < 0.05; **p < 0.01; ***p < 0.001. p values are based on Wilcoxon tests. [file 12920_2022_1196_MOESM6_ESM.tif]

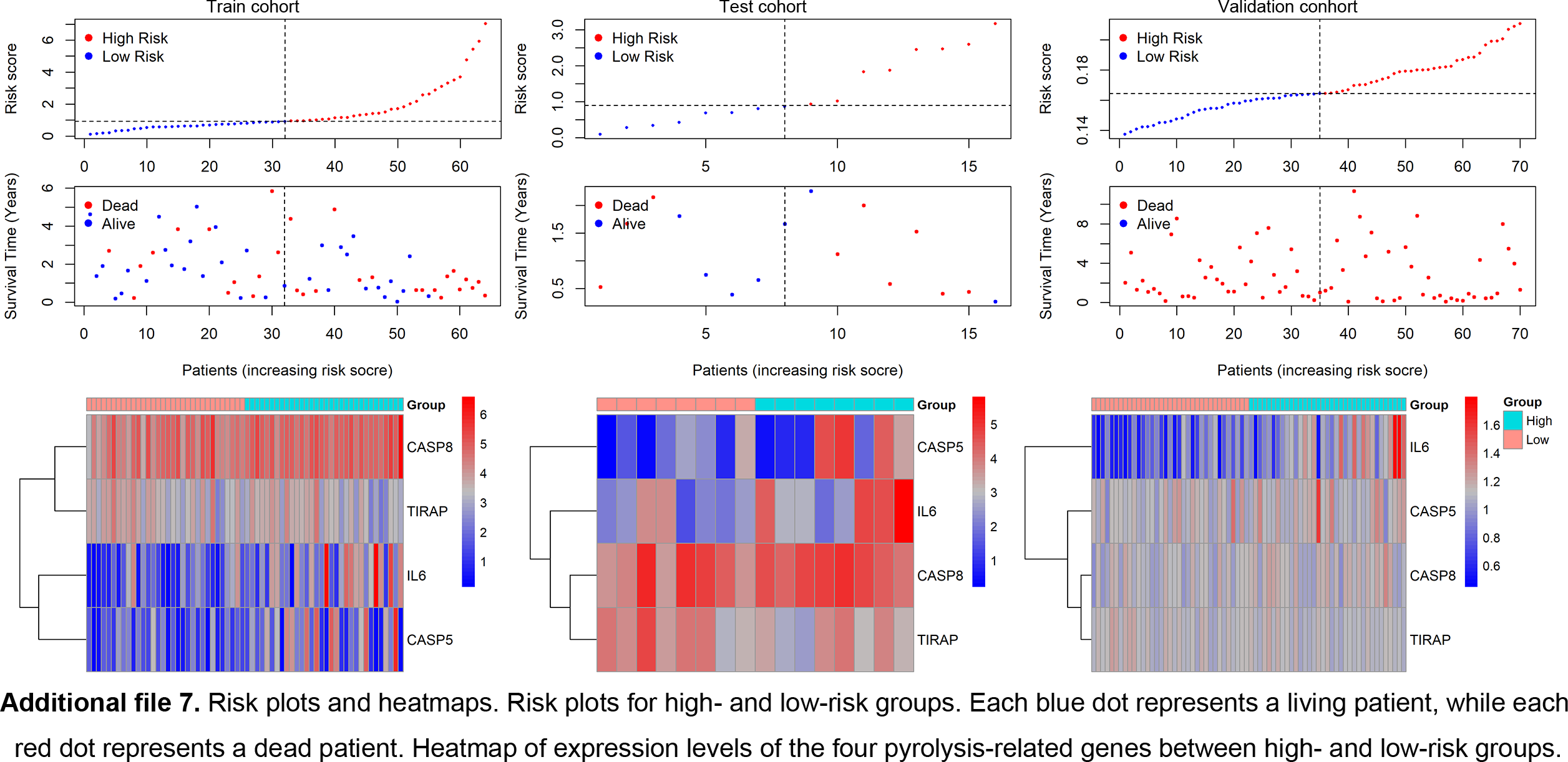

Supplement: Supplementary file 7 — Additional file 7. Risk plots and heatmaps. Risk plots for high- and low-risk groups. Each blue dot represents a living patient, while each red dot represents a dead patient. Heatmap of expression levels of the four pyrolysis-related genes between high- and low-risk groups. [file 12920_2022_1196_MOESM7_ESM.tif]

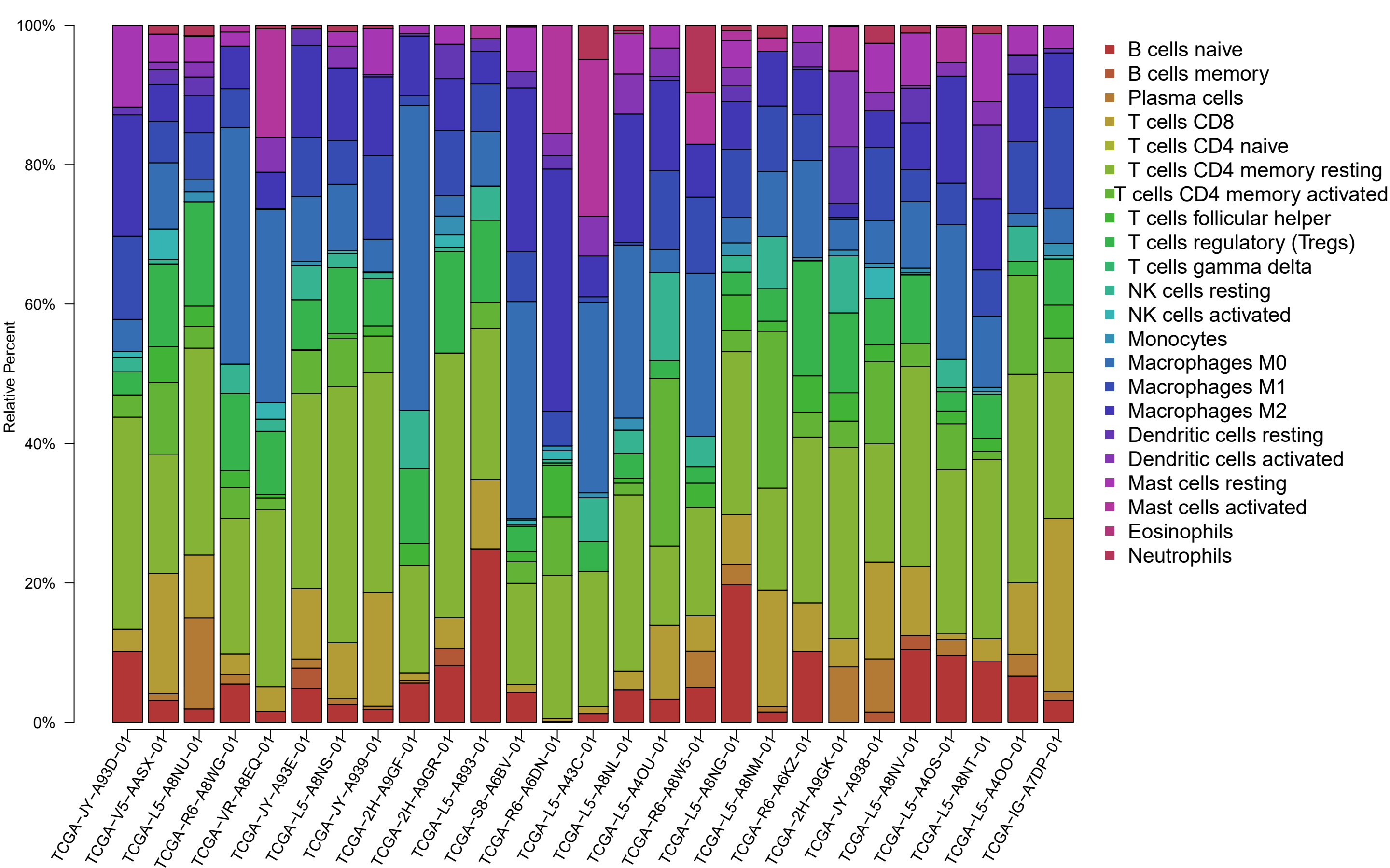

**Additional file 9.** Immune cell infiltration in high-risk and low-risk groups for each sample.

Supplement: Supplementary file 9 — Additional file 9. Immune cell infiltration in high-risk and low-risk groups for each sample. [file 12920_2022_1196_MOESM9_ESM.pdf]

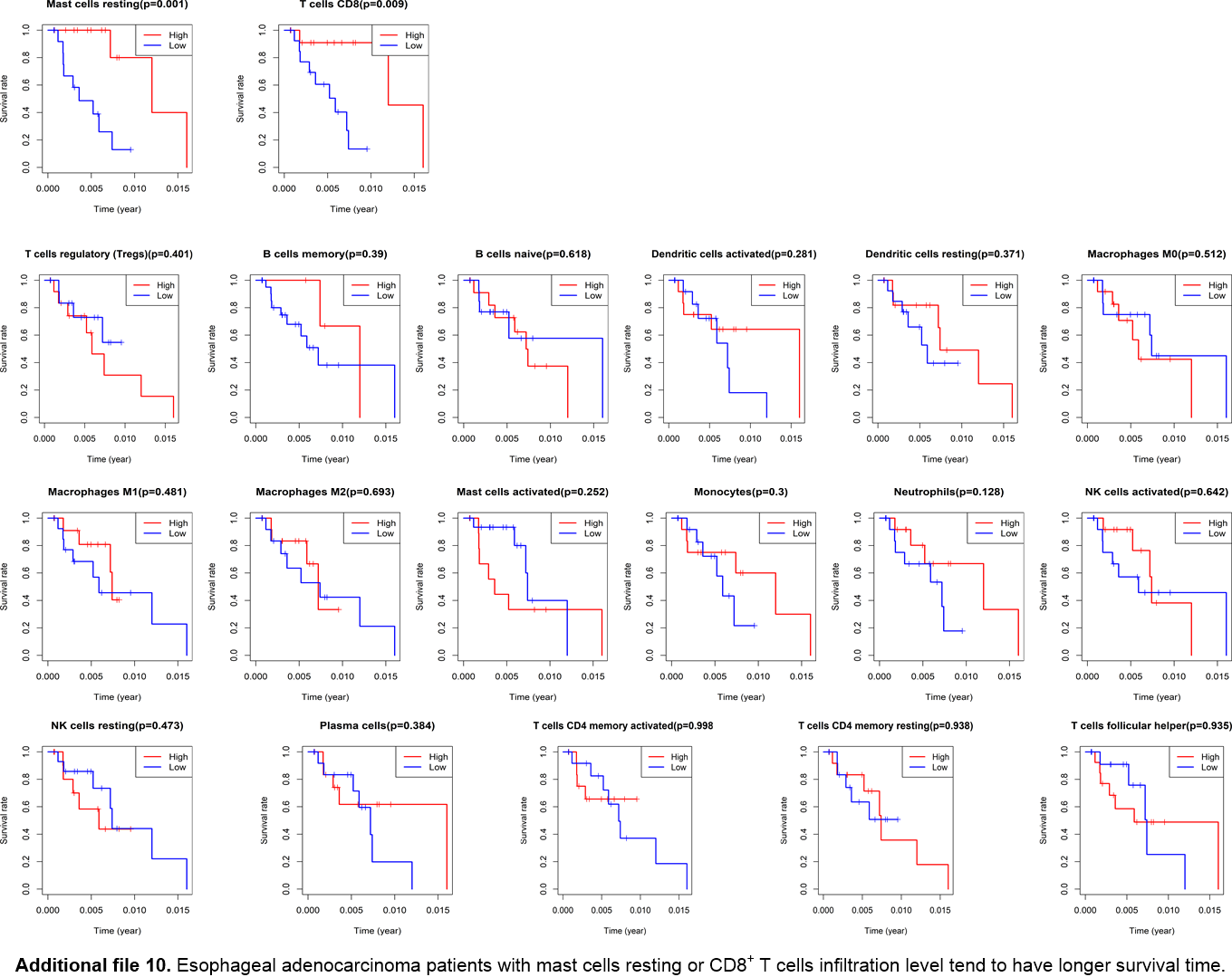

Supplement: Supplementary file 10 — Additional file 10. Esophageal adenocarcinoma patients with mast cells resting or CD8+ T cells infiltration level tend to have longer survival time. [file 12920_2022_1196_MOESM10_ESM.tif]

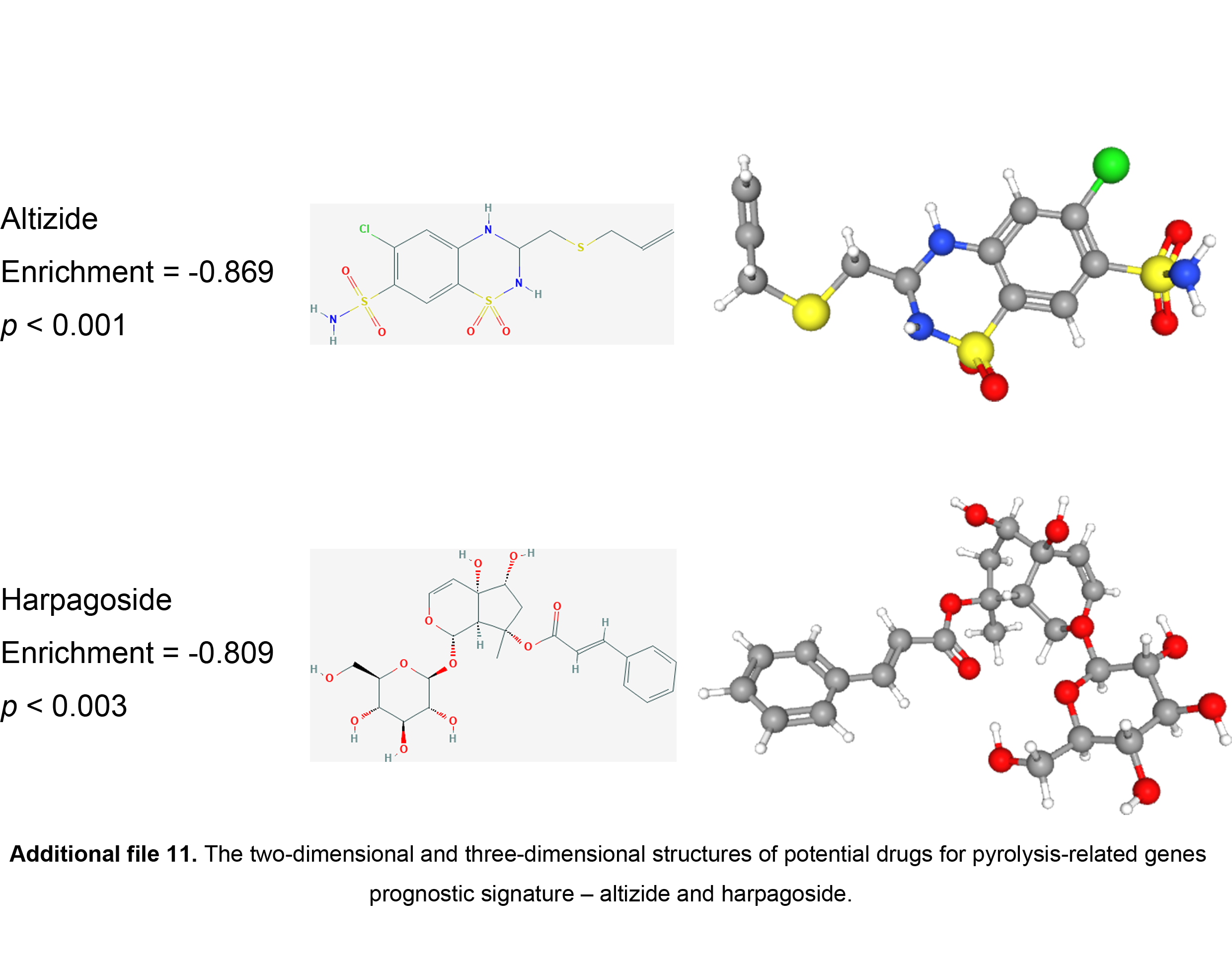

Supplement: Supplementary file 11 — Additional file 11. The two-dimensional and three-dimensional structures of potential drugs for pyrolysis-related genes prognostic signature – altizide and harpagoside. [file 12920_2022_1196_MOESM11_ESM.tif]
