# Supplementary material for: Prognostic signature of esophageal adenocarcinoma based on pyroptosis-related genes
Source: BMC Med Genomics. 2022 Mar 7;15:50. doi: 10.1186/s12920-022-01196-x (PMC8900411; doi:10.1186/s12920-022-01196-x)
Supplement: Supplementary file 2 — Additional file 2. The data sets included in this study. [file 12920_2022_1196_MOESM2_ESM.docx]

**Additional file 2.** The data sets included in this study.

| **Platform** | **Data set** | ***n* of samples** | |  | **Platform** | **Data set** | ***n* of samples** | |
| --- | --- | --- | --- | --- | --- | --- | --- | --- |
|  |  | **EAC** ^a^ | **NE** ^b^ |  |  |  | **EAC** | **NE** |
| GPL2507 | GSE28302 | 23 | 9 |  | GPL6102 | GSE13898 | 75 | 28 |
| GPL10558 | GSE72873 | 48 | 17 |  | GPL570 | GSE26886 | 21 | 19 |
|  | GSE93352 | 3 | 0 |  |  | GSE64894 | 0 | 3 |
|  | GSE47763 | 3 | 0 |  |  | GSE42363 | 14 | 0 |
|  | GSE57130 | 3 | 3 |  |  | GSE9974 | 3 | 0 |
| GPL17692 | GSE74553 | 52 | 8 |  |  | GSE7964 | 1 | 0 |
|  | GSE77563 | 0 | 40 |  |  | GSE7307 | 0 | 4 |
| GPL6244 | GSE92396 | 12 | 10 |  |  | GSE148247 | 0 | 3 |
|  | GSE100843 | 0 | 18 |  |  | GSE45670 | 0 | 10 |
|  | GSE34619 | 0 | 8 |  |  | GSE27424 | 0 | 6 |
|  | GSE36725 | 0 | 5 |  |  | GSE17353 | 0 | 4 |
| GPL96 | GSE37200 | 15 | 0 |  |  | GSE3526 | 0 | 4 |
|  | GSE1420 | 8 | 8 |  |  | GSE19472 | 0 | 2 |
|  | GSE13083 | 0 | 7 |  |  | GSE33810 | 0 | 1 |
|  | GSE52138 | 0 | 2 |  |  | GSE100942 | 0 | 5 |
|  | GSE23400 | 0 | 53 |  |  | GSE17351 | 0 | 5 |
|  | GSE44021 | 0 | 34 |  |  | GSE77861 | 0 | 7 |
| TCGA-GTEx | TCGA ^c^ | 80 | 10 |  |  | GSE161533 | 0 | 56 |
|  | GTEx ^d^ | 0 | 291 |  |  | GSE44021 | 0 | 6 |

Notes: ^a^: esophageal adenocarcinoma; ^b^: normal esophagus; ^c^: the Cancer Genome Atlas; ^d^: the Genotype-Tissue Expression.
